# Untargeted histone profiling during naive conversion uncovers conserved modification markers between mouse and human

**DOI:** 10.1038/s41598-019-53681-6

**Published:** 2019-11-21

**Authors:** Laura De Clerck, Jasin Taelman, Mina Popovic, Sander Willems, Margot Van der Jeught, Björn Heindryckx, Petra De Sutter, Hendrik Marks, Dieter Deforce, Maarten Dhaenens

**Affiliations:** 1 ProGenTomics, Laboratory of Pharmaceutical Biotechnology, Ghent University, Ottergemsesteenweg 460, 9000 Ghent, Belgium; 2 Ghent-Fertility and Stem Cell Team (G-FaST), Department for Reproductive Medicine, Ghent University Hospital, Corneel Heymanslaan 10, 9000 Ghent, Belgium; 3 Department of Molecular Biology, Faculty of Science, Radboud University, Radboud Institute for Molecular Life Sciences (RIMLS), 6525GA Nijmegen, the Netherlands

**Keywords:** Embryonic stem cells, Epigenetics, Proteomics, Post-translational modifications

## Abstract

Recent progress has enabled the conversion of primed human embryonic stem cells (hESCs) to the naive state of pluripotency, resembling the well-characterized naive mouse ESCs (mESCs). However, a thorough histone epigenetic characterization of this conversion process is currently lacking, while its likeness to the mouse model has not been clearly established. Here, we profile the histone epigenome of hESCs during conversion in a time-resolved experimental design, using an untargeted mass spectrometry-based approach. In total, 23 histone post-translational modifications (hPTMs) changed significantly over time. H3K27Me3 was the most prominently increasing marker hPTM in naive hESCs. This is in line with previous reports in mouse, prompting us to compare all the shared hPTM fold changes between mouse and human, revealing a set of conserved hPTM markers for the naive state. Principally, we present the first roadmap of the changing human histone epigenome during the conversion of hESCs from the primed to the naive state. This further revealed similarities with mouse, which hint at a conserved mammalian epigenetic signature of the ground state of pluripotency.

## Introduction

Human embryonic stem cells (hESCs) can be maintained indefinitely *in vitro*. This capacity for self-renewal allows harnessing their intrinsic capability of pluripotency, the ability to differentiate into any somatic cell type^1–3^. However, pluripotency is a product of the cellular state in which hESCs reside, which is directly shaped by the culture microenvironment^4–7^. Conventional derivation conditions generate hESCs that are transcriptionally in a primed state of pluripotency. This state is more similar to the *in vivo* post-implantation epiblast, as opposed to the preimplantation epiblast from which hESCs are derived^8,9^. In contrast, mouse ESCs (mESCs) conventionally reside in the naive state of pluripotency, which maintains high resemblance to the preimplantation epiblast^10^. As such, mESCs remain the accepted paradigm of ground state pluripotency^11^.

Compared to naive mESCs, primed hESCs are more prone to lineage specification bias and ultimately culture heterogeneity^10,12–14^. In an effort to address these shortcomings, several groups have succeeded in formulating culture environments that convert primed hESCs into a more naive state, albeit with varying sets of naive traits^11,15–17^. The different protocols used to generate naive hESCs have provided many insights into the transcriptional landscape and the DNA methylation status of human naive pluripotency^11,14,18,19^. However, these diverse naive protocols have also raised uncertainty over true naive hallmarks^11^. Currently, preferential use of distal over proximal enhancer elements to induce expression of *POU5F1*, a global decrease in CpG DNA methylation and reactivation of one X-chromosome in female hESC lines are the most prominent, generally accepted features of naive pluripotency^11^. However, overall, the epigenetic differences that underlie primed and naive states remain unclear, particularly regarding chromatin modifications^18^.

Histone post-translational modification (hPTM) patterns are distinct between naive and primed hESCs^18^. However, no comprehensive picture of the changing hPTM landscape has been described thus far. At least in part, this is because the knowledge on histone epigenetics is built on individual antibody-based assays, such as western blot and chromatin immunoprecipitation (ChIP), targeting only a single hPTM per assay. Therefore, when applying these techniques, a restricted list of target hPTMs needs to be selected. Thereby, ChIP-based assays have demonstrated that primed hESCs exhibit an increase in bivalent chromatin domains on lineage regulatory gene promotors, characterized by the deposition of the repressive histone mark, trimethylation of lysine 27 of histone H3 (H3K27Me3) and the activating histone mark H3K4Me3^19–22^. Conversely, multiple naive culture conditions have been shown to cause a reduction in H3K27Me3 marks over developmental genes compared to primed conditions, suggesting a more accessible chromatin state in naive hESCs^11,19,20^. This is in agreement with what is known for mESCs^23^. In a developmental context, the prevailing view is that a gradual increase in repressive histone marks occurs during continued developmental progression from naive to primed pluripotency and ultimately differentiation into somatic cell types, binding a cell to its fate^11^.

At present, antibody-based assays are increasingly complemented with mass spectrometry (MS)-based strategies for the study of the histone epigenome^24,25^. Untargeted MS-based approaches create a very different and ultimately more comprehensive perspective that comprises several dozens of genome-wide hPTM abundances in a single assay. In fact, it is no longer considered expedient to confirm MS data with antibody assays, due to the many potential artefacts associated with antibodies directed to hPTMs^26,27^. In the past years, MS-based approaches have not only revealed new hPTMs, but have also provided combinatorial information, generating a more complete picture of the histone code^24,28–30^. Recently, by using a bottom-up MS approach, the polycomb repressive complex 2 (PRC2) and the H3K27Me3 mark it catalyzes were unexpectedly identified as the most abundant features that were significantly increased in naive (2i) mESCs as compared to primed (serum) mESCs^31^. This illustrates the importance of creating a bird’s eye view of the histone code as a framework, before antibody-based approaches are used to localize the hPTMs over the genome.

In this study, we set out to picture the dynamics of the histone epigenome during the conversion of primed to naive hESCs using an untargeted MS-based assay. For this, we sampled the conversion in a feeder-free culture system every three passages over 12 passages, i.e. 37 days. This time resolution enabled the characterization of many transient changes in both protein expression and hPTM abundance, in line with a staged transition process. In search of naive hPTM markers, we further compared passage 0 (P0) and passage 12 (P12) directly. Herein, H3K27Me3 was the most prominently increased hPTM genome-wide in naive compared to primed hESCs, in line with our recent findings in mouse^31^. As the same MS-assay was used in both studies, we had the unique opportunity to further investigate all hPTM changes between the primed and naive state, in mouse and human. This comparison revealed a robust set of naive markers that are conserved between the two species. Notably, we provide the first roadmap of changes in the hESC histone fingerprint during the conversion from primed to naive pluripotency.

## Results

### Conversion from primed to naive hESCs is a staged process

**Figure 1:**
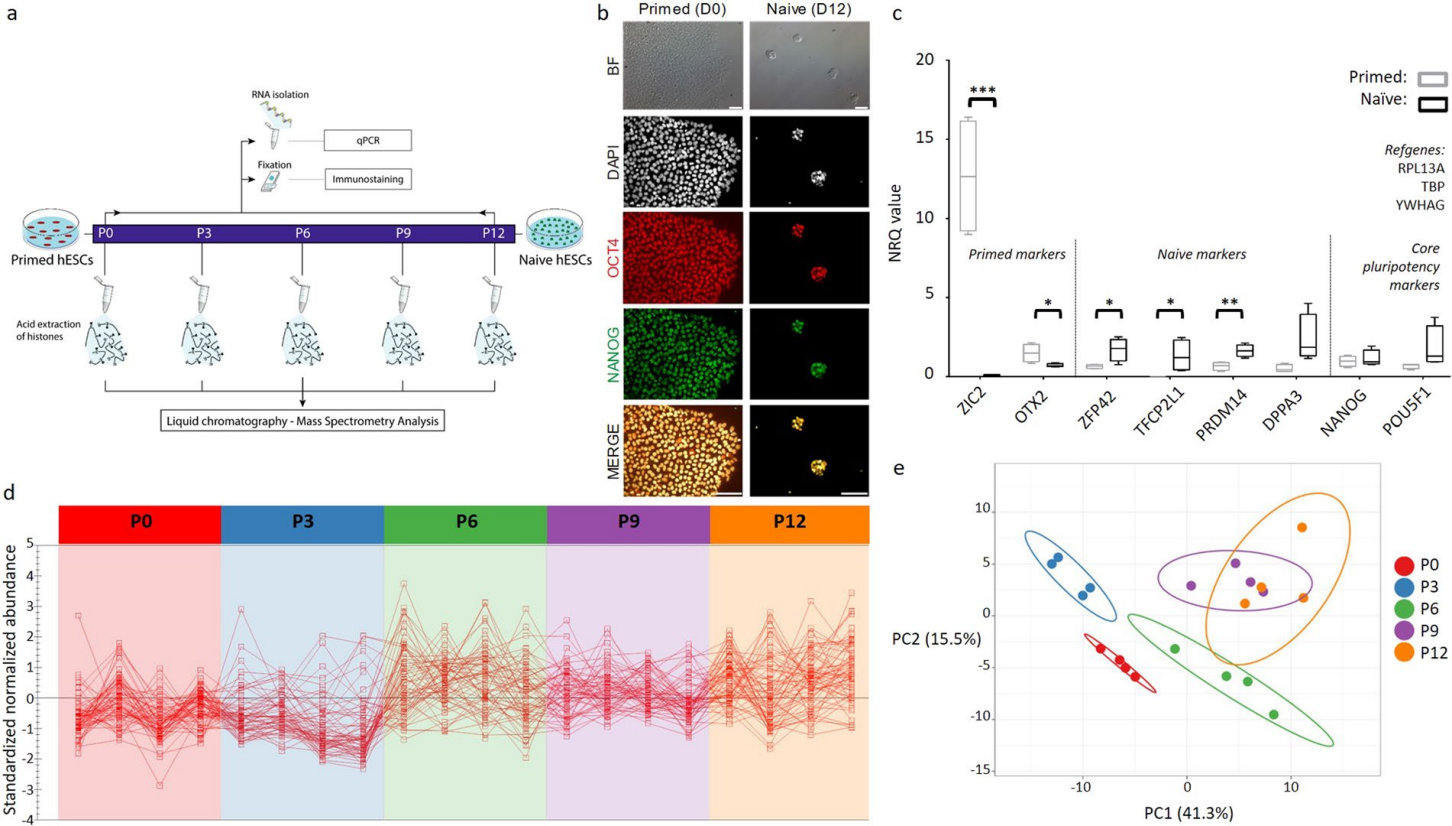
Conversion of primed (P0) to naive (P12) hESCs. (**a**) Time-resolved experimental design used for sampling. hESCs were harvested at five different passages (P0-P3-P6-P9-P12), each in four biological replicates. (**b**) Light and fluorescence microscopy images of primed (P0, left) and naive (P12, right) hESCs. P12 colonies became domed, with clear OCT4 (*POU5F1*) and NANOG expression. BF = brightfield. Scalebar = 100 µm (**c**) NRQ values (Normalized Relative Quantitative cycle (Cq) values) of the qPCR analysis of 8 different pluripotency markers in primed (grey) and naive (black) hESCs (N = 4; *p < 0.05; **p < 0.01; ***p < 0.001). **(d)** Standardized normalized abundances of the 64 translation-associated proteins in each individual replicate are depicted and connected with a red line to highlight trends and biological variation between replicates (N = 4). The protein abundances steeply increase between P3 and P6 (*p* = 2.79E-06, two-tailed paired student’s t-test between the average abundances). **(c)** PCA of the acid extractome throughout the conversion. Differential protein abundances of the acid extractome cluster P3 markedly away from other time points in Principle Component 1 (PC1). P9 and P12 clusters overlap, implying that a stable naive state is reached. Prediction ellipses are such that with probability 0.95, a new observation from the same group will fall inside the ellipse. See also Supplementary Fig. S1, Supplementary Tables S2 and S3.

To evaluate the naive conversion, we primarily compared primed (P0) and naive hESCs (P12) cultured in feeder-free Enhanced Weizmann Institute of Science – Naive Human Stem cell Medium (WIS-NHSM) conditions (Fig. 1a)^20^. Following the naive conversion, colonies became dome-shaped and were positive for OCT4 (*POU5F1*) and NANOG (Fig. 1b). No difference in pluripotency markers *POU5F1* (p = 0.134) and *NANOG* (p = 0.605) expression was observed between primed and naive hESCs, while expression of naive markers *DPPA3* (p = 0.054), *PRDM14* (p = 0.005), *TFCP2L1* (p = 0.0395) and *ZFP42* (p = 0.0276) was significantly increased in naive compared to primed hESCs (Fig. 1c and Supplementary Table S1). Conversely, primed markers *OTX2* (p = 0.035) and *ZIC2* (p = 0.0005) were significantly reduced in naive hESCs compared to primed counterparts (Fig. 1c and Supplementary Table S1).

Furthermore, a set of quantifiable proteins were (co-)extracted and identified during the histone acid extraction (Supplementary Table S2). In conventional bottom-up proteomics, this unique subset of the proteome, which we refer to as “the acid extractome” is not usually covered. These proteins are basic in nature, i.e. do not precipitate in acid (Supplementary Fig. S1) and were identified following chemical derivatization (propionylation) prior to tryptic digestion and display ArgC specificity. We performed gene ontology (GO) analysis to understand the potential biology harboured in this unique subset of the proteome (Supplementary Fig. S1). The upregulation of proteins associated with GO terms “citric acid cycle (CAC), respiratory electron transport” (R-HAS-1428517) and “Translation” (R-HSA-72766) at P12 is in line with the well-established metabolic shift and increased protein synthesis rate in the naive state, respectively^11^ (Supplementary Fig. S2a). Additionally, the most significant protein fold changes correlated strongly with mRNA fold changes described in 5 inhibitors + LIF + Activin A (5iLA) conditions^21^ (Supplementary Fig. S2a,b). Upregulated proteins in our naive hESCs comprised: (i) ZNF593 (*mRNA in 5iLA: Log2FC* = *1.926, p* = *0.003*), (ii) EDF1 (*mRNA in 5iLA: Log2FC* = *1.063, p* = *0.039*), (iii) TIMM44 (*mRNA in 5iLA: Log2FC* = *1.882, p* = *0.0069*), (iv) DDX27 (*mRNA in 5iLA: log2FC* = *1.2173, p* = *0.0296*), (v) COX5A (*mRNA in 5iLA: Log2FC* = *1.230, P* = *0.019*) and (vi) NOP16 (*mRNA in 5iLA: log2FC* = *2.1685, p* = *0.0343*). NOCL3L, CC137, RS27A/RL40, RL35, UCRIL and NH2L1 were not detected at the mRNA level in Theunissen *et al*., but were equally upregulated at the protein level in the acid extractome. Downregulated proteins in our naive hESCs included: (i) CBX5 (*mRNA in 5iLA: Log2FC* = −*1.909, p* = *0.00075*) and (ii) the chromatin binding protein NUCKS1 (*mRNA in 5iLA: log2FC* = −*0.059, p* = *0.800*) (Supplementary Fig. S2b). Accordingly, our data revealed a similar profile to the mRNA fold changes between naive and primed hESCs described by Theunissen *et al*. in all but one protein: NUCKS1, which was not differentially expressed in Theunissen *et al*.^21^. Overall, we demonstrate that the feeder-free hESCs cultured for 12 passages were in a naive state of pluripotency.

The time-resolved sampling of the acid extractome also allowed us to examine the process of conversion. A total of 154 proteins of the acid extractome had an ANOVA Q-value < 0.01. The 64 translation-associated proteins, most prominently the 33 ribosomal L-subunits, steeply increased between P3 and P6, suggesting that cell proliferation increased between these timepoints (Fig. 1d). Interestingly, principle component analysis (PCA) of all differential proteins did not show directionality according to the timeline of the experiment (Fig. 1e). Rather, P3 clustered at the negative side of the first principle component, implying that it was substantially different from the other time points in terms of protein expression. Moreover, an important histone epigenetic mediator, histone deacetylase 2 (HDAC2), was transiently downregulated at P3 (Supplementary Table S2). Overall, this indicates that the initial stimulation causes a profound disturbance in the acid extractome and by extension the proteome and cell phenotype. Accordingly, following P3 we observed domed-shaped colonies within the culture for the first time, a distinctive feature of the naive state. On the other end of the PCA, the P9 and P12 clusters overlapped, indicating that these cells reached a stable, naive state (Fig. 1e).

Finally, significant changes were found in histone variants and linker histones (Supplementary Fig. S2c). Specifically, histone H2A variants H2AW (ANOVA Q-value = 4.51e-8) and H2AY (ANOVA Q-value = 1.40e-07) showed a brief increase in expression at P3, after which they disappeared almost entirely. Histone H1 variant H11 was the most differentially abundant protein in the acid extractome (ANOVA Q-value = 1.66e-10) and was only detected in P9 and P12, at the cost of the other H1 variants (Supplementary Fig. S2c).

Taken together, this time-resolved protein analysis suggests that hESCs are not linearly transformed, but rather transition through different phases of molecular changes from the primed to the naive state of pluripotency.

### The histone code is highly dynamic during conversion

Mapping of all the identified histone peptidoforms against all known validated histone modifications as previously described^32^, allowed identification and quantification of 128 hPTM combinations on histone H3 and H4 (Supplementary Table S3). These peptidoforms were used to calculate the relative abundance (RA) for 49 individual hPTMs. With four biological replicates, this is equivalent to using 49 different antibody assays on 20 different samples, but with considerably higher quantitative accuracy. Furthermore, the different peptidoforms comprise peptides with a single modification, with multiple modifications, and unmodified peptides, adding an additional level of combinatorial information compared to antibody-based assays. However, by breaking up the histone protein backbone, variant calling becomes possible only if the distinctive amino acid occurs on the peptidoform carrying the hPTM. Generally, accurate quantification of histone H3 variants is more complicated than e.g. H2A variants, as they vary very little from one another^33^. This is best illustrated for histone H3 variant 1 (H31)/histone H3 variant 2 (H32) compared to histone H3 variant 3 (H33), which can be distinguished by the substitution of A31 to S31 that is embedded in the sequence stretch KSAPATGGVKKPHR, which also covers K27, K36 and K37. For H31 and H32, no such amino acid substitution was covered in our analysis, therefore we depicted them interchangeably. When no variant could be called at all, we just depicted H3. Inversely, if two PTMs are on neighboring residues, fragmentation spectra often lack the one fragment needed to localize it to one of the two. For example, to avoid misinterpretation, we depicted K36 and K37 as interchangeable, i.e. K36/37.

**Figure 2:**
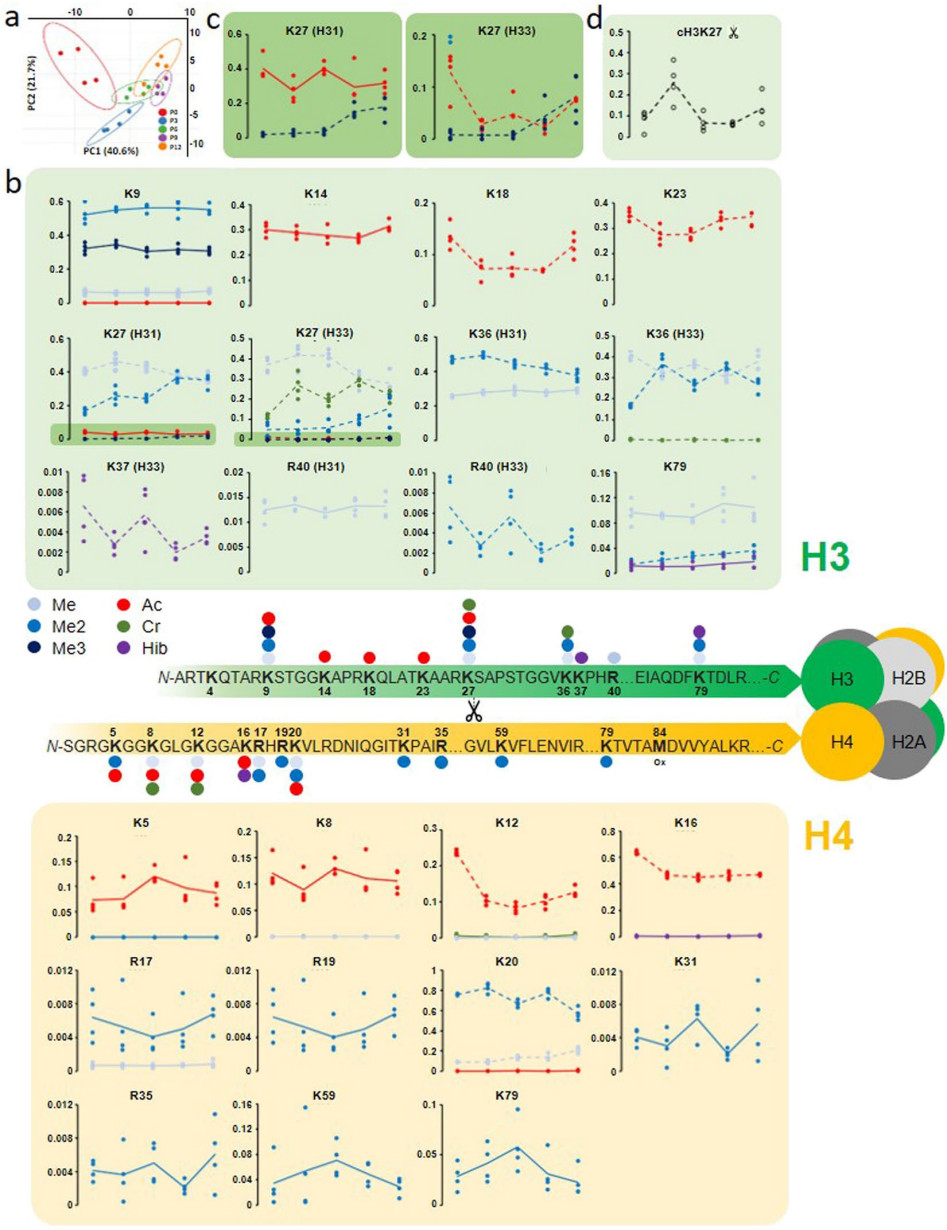
The hPTM dynamics of histone H3 and H4 during the conversion of primed to naive hESCs. (**a**) PCA of the differential histone peptidoforms used to calculate the RA of single hPTMs throughout the conversion. Differential histone peptidoform abundances cluster P0 markedly away from other time points in PC1. P6, P9 and P12 clusters overlap, implying that histones stabilize faster than the protein expression profile. Prediction ellipses are such that with a probability of 0.95, a new observation from the same group will fall inside the ellipse. See also Supplementary Table S3 for the normalized histone peptide abundanes. (**b**) A comprehensive map of the relative abundance (RA) of hPTMs (in the Y-axis) on 12 residues of histone H3 variants and 11 residues of histone H4 over 12 passages, i.e. a 37 day transition experiment from primed to naive hESCs. The X-axis representing the different passages (P0-P3-P6-P9-P12) was omitted for clarity. Individual RA measurements are depicted by dots and their averages over four biological replicates are connected by a line. Each graph represents a location and the coloured lines depict the different hPTMs that were quantified on that residue. hPTMs that are significantly changing over time, i.e. with an ANOVA *P*-value < 0.05, are depicted by dotted lines, while full lines are statistically stable. The RA of the unmodified peptides are not depicted, but simply accounted for by the remaining RA (adding up to 100%). (**c**) A zoom with scaled Y-axis for H31K27 and H33K27, to highlight the significant changes seen in the low RA region, i.e. <5% RA. See also Supplementary Table S4 for the pairwise *P-*values between all time points. (**d**) cH3K27: RA of the histone clipping event at H3K27 (depicted by the scissors icon in (**b**)).

In the PCA of the differential histone peptidoform abundances (N = 84), P0 clustered away from the rest of the passages (Fig. 2a), unlike P3, as observed in the acid extractome analysis (Fig. 1e). Coupled to the fact that P6, P9 and P12 all clustered together, this suggests that hPTMs also change most drastically upon stimulation, but stabilize faster than the proteins within the acid extractome.

As expected, many hPTMs changed significantly throughout the conversion (Fig. 2b, Supplementary Table S4). Some of the significant changes were transient and only appeared during an intermediate passage, after which they return towards their initial levels. These hPTMs can thus not serve as naive markers, but they are most probably pivotal in driving the genome-wide histone epigenome changes that take place during conversion from primed to naive hESCs. The most prominently increased hPTM was H3K27Me3 (Fig. 2c), which was abruptly elevated at P9 on both H31 and H33. This coincided with the point at which a stable naive state was reached according to the acid extractome (Fig. 1e). Notably, acetylation (Ac) showed an overall decline upon stimulation, i.e. from P0 to P3, after which it was partially restored towards the stable naive state. This trend was significant on H3K18, H3K23, H33K27, histone H4 lysine 12 (H4K12) and H4K16 (Fig. 2b).

Additionally, untargeted MS allowed detection of histone clipping events, which have been hypothesized to play an important role in stem cell biology^34^. We only depicted clipped histone H3 lysine 27 (cH3K27), which was examined in the greatest detail (Fig. 2d). The highest abundance of this clipping event was measured at P3, to around 3% of histone H31 genome-wide.

### Histone markers of the naive and primed state are conserved in human and mouse

**Figure 3:**
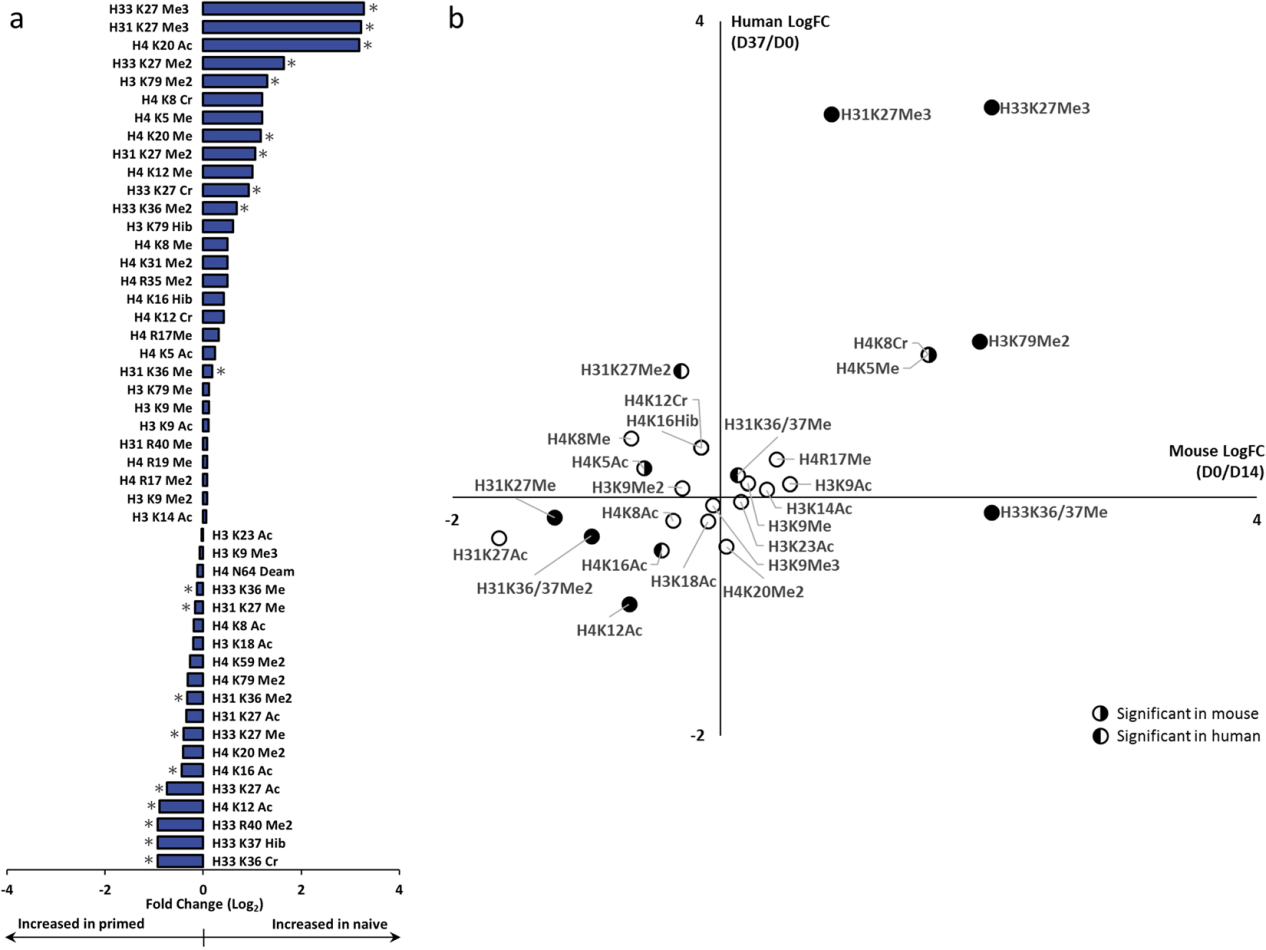
Conserved histone markers of the naive and primed state in human and mouse. (**a**) Relative abundance (RA) fold changes between primed (P0) and naive (P12) hESCs. Asterisks indicate significance (N = 4, *p* < 0.05). (**b**) Scatter plot of the log2 fold changes of the RA of hPTMs between the naive and primed state, which were identified in both hESCs and mESCs. Significant changes in mouse are indicated by a right hemisphere, while the left hemisphere indicates significance in human. Dx: Day x of the experiment. mESC were converted from naive to primed and hESCs from primed to naive. Histone H31 and H33 as well as K36 and K37 were merged for this comparison. H4K8Cr and H4/K5Me were identified together on the same peptide backbone and thus have identical RA values, in both mouse and human.

Our analysis revealed 21 hPTMs with a significant fold change in RA between naive (P12) and primed (P0) hESCs (T-test *p* < 0.01) (Fig. 3a, Supplementary Table S5). We consider these to be potential marker hPTMs of the naive and primed state. The most prominently increased hPTMs in naive cells are di- and trimethylation on K27 of H31 and H33. Additionaly, histone H3 variant 3 lysine 27 crotonylation (H33K27Cr), H31K36Me/H33K36Me2, H3K79Me2 and H4K20Ac/H4K20Me were more abundant in naive cells. On the other hand, potential primed markers are H33K27Ac/H31K27Me/H33K27Me, H33K36Me/H31K36Me2/H33K36Cr, histone H3 variant 3 lysine 37 2-hydroxyisobutyrylation (H33K37Hib) and H33R40Me2, together with H4K12Ac and H4K16Ac.

In agreement with our recent findings reported in mouse^31^, we reveal a genome-wide increase of H3K27Me3 as a hallmark of naive hESCs. This prompted us to extend the comparison of potential hPTM marks to all the common fold changes detected in both studies, allowing us to generate a list of conserved hPTM marker candidates. However, our hESCs were converted from a primed to naive state in 37 days, while the mESCs were previously converted from a naive to a primed state in 14 days^31^. Therefore, we expressed hPTM changes over time as day 37/day 0 (D37/D0) for hESCs and as day 0/day 14 (D0/D14) for mESCs. (Fig. 3b). We were able to quantify 27 hPTMs based on RA, in both studies (Fig. 3b).

The fact that naive mESCs were converted into the primed state by changing 2i media to serum-containing media over 14 days^31^, while here, primed hESCs were converted to the naive state over 37 days using Enhanced WIS-NHSM conditions, emphasizes the robustness of this set of hPTMs in ESCs. Despite the inverse timeline, the most prominent differentially detected hPTMs behaved strikingly similar between mouse and human, in both naive and primed states. In some cases they were equally significant. Moreover hPTMs that did not contribute significantly to either state were also shared between mouse and human. Therefore, common changes identified through unbiased comparison of all annotatable histone PTMs can only be explained by the state the cells reside in. The most striking outliers were H31K27Me2, which was upregulated in human and not in mouse, as well as H33K36/37Me, which was upregulated in mouse and not in human.

Taken together, our untargeted MS assay on the conserved hPTMs, allowed direct comparison of the histone epigenome between these two evolutionarly distinct species, for the first time. The significant overlap between the changes that occur from the primed to the naive state, irrespective of the developmental timeline, illustrate that hPTMs hold great promise as naive markers.

## Discussion

We present a first comprehensive histone epigenome roadmap of the conversion of primed hESCs to the naive state of pluripotency. We employed an untargeted MS-based approach with a time-resolved experimental design. By sampling the conversion process itself, changes in the histone epigenome can be investigated in a more causal context, correlating changing hPTMs to a time axis. This is a feature that is absent in marker discovery, where binary comparisons are standard. To our knowledge, no such time-resolved untargeted monitoring of the histone fingerprint has been performed during any stem cell conversion.

We validated our naive hESCs, not only using conventional methods including morphology and marker expression, but also by employing novel mass spectrometry approaches. The acid extractome is co-extracted in the process of purifying histones. However, the histones are merely a selection of targets in a proteomic approach. By its nature, the protocol intrinsically enriches basic proteins. These proteins are basic for functional reasons, as they need to interact with negatively charged nucleotides in the cell. Analysis of the acid extractome revealed an increased translational protein load, as well as elevated CAC proteins, which are commonly associated with naive pluripotency^11^. Moreover, all but one (NUCKS1) of the statistically different proteins in our acid extractome matched the differentially expressed mRNA in the microarray data from an entirely different, but widely accepted naive condition^21^. Finally, by monitoring the conversion over time, a PCA of the protein expression patterns illustrated that the hESCs reached a stable state prior to the end of the experiment, at around P9.

Recently, it was suggested that an intermediate stage of pluripotency exists during the transition from the naive to the primed state in a human developmental context, namely the formative state^35,36^. Whether this formative state is also detectable in reversed biological order *in vitro*, has not been described. Interestingly, our data suggest that the conversion may be a staged process. More specifically, we observed P3 to significantly differ from all other passages. Many of the proteins transiently decreased in expression at P3 after which they return to original levels, including HDAC2. Notably, it was recently shown that transient chemical inhibition of HDAC2 can be used as a method to convert primed hESCs into a more naive state^37^. Conversely, H2A variant proteins H2AY and H2AW were not transiently downregulated at P3, but were instead briefly increased, after which they completely disappeard towards P12. Interestingly, these “macroH2A” variants are known to be enriched in chromatin on the inactive X-chromosome of female cells. We further identified 67 translational proteins which all showed a sudden increase in expression between P3 and P6. We interpret this as the stage in which cell proliferation accelerates, which in turn relies on protein synthesis upregulation. Accordingly, this was also the timepoint at which the first dome-shaped colonies appeared.

Markedly, histone peptidoforms also change most drastically at P3. Yet, they stabilize more quickly compared to the proteins, in line with their role in transcriptional regulation. In total, 23 different hPTMs were influenced by the treatment over time. Our histone epigenome roadmap thus provides a target list of hPTMs that change on a genome-wide scale. This list also includes the clipping of histone H3 at K27 (cH3K27)^34^. cH3K27 is known to play a role in both mouse and human stem cell differentiation, but was also recently shown to be enriched in naive 2i cultured mESCs^31,38,39^. These time-resolved experimental designs have shown that the clipping is highest upon stimulation (irrespective of the compound used) and disappears later on. Taken together, our results and those of others suggest that histone clipping provides a way for the cell to “reboot” the histone epigenome upon stimulation, possibly independent of the effect of the stimulation.

It is widely accepted that conventional mESCs are the prototype of naive pluripotency^11^. Accordingly, many of the most prominent hPTM fold changes between primed and naive hESCs were consistent with the changes which we recently reported in mouse^31^. As an identical workflow in terms of sample preparation, data acquisition and data analysis was applied in both studies, we had the unique opportunity to compare the MS-based results from hESCs to those from mESCs. Markedly, this revealed a set of conserved naive hPTM marker candidates.

The fact that mESCs and hESCs were converted in a reverse direction in the two studies emphasizes the robustness of this set of hPTMs for the naive state of pluripotency. The most prominent changes that were found in both species on histone H3 variants, included increased trimethylation at K27Me3 and dimethylation at K79. Interestingly, H3K79Me2 has been shown to promote proliferation, which is generally accepted to be increased in naive cells^11^. On histone H4, monomethylation at K5 and crotonylation at K8 co-increased on the same peptide backbone in both mouse and human. Conversely, we observed a decrease in demethylation of H31K36, as well as decreased acetylation at H4K12 and H4K16 in both species, as the cells transitioned towards the naive state. Remarkably, dimethylation at H3K27 was an outlier, being significantly increased in hESCs, while decreased in mESCs, albeit not significantly. This may indicate species-specific differences in the use of this specific hPTM. Taken together, we provide a bird’s eye view of the changing hPTM landscape in both human and mouse ESCs and present a valuable list of naive marker candidates.

In previous studies on primed and naive hESCs the primary experimental approaches used for profiling hPTMs are antibody-based assays (e.g. ChIP-Seq and western blot), allowing the profiling of only a limited number of hPTMs. The most targeted hPTMs are H3K4Me, H3K4Me3, H3K9Me3, H3K27Me3, and H3K27Ac. The H3K4 peptides are poorly recovered by bottom-up MS because they are not well retained on C18 liquid chromatography colums^40^. The H3K9Me3 mark was shown to be depleted in naive cells and cells transitioning towards the primed state^20,41,42^. Similarly, we detected a decrease of H3K9Me3 in the naive state, although it was not significant. In contrast to our results however, previous studies using regular ChIP-Seq have reported higher levels of H3K27Me3 in primed hESCs, compared to naive hESCs^20,21,41,42^. Notably, it has been shown that quantitative analysis of ChIP-Seq signals require the use of a spike-in for proper normalization^31^. This equally holds for H3K27Ac^41^, which is mutually exclusive with H3K27Me3. Finally, western blot assays can only be compared to our results when normalization is done similarly, i.e. against total histone content. One such example indeed confirms that overall H3K27Me3 increases towards the naive state^43^.

Overall, untargeted MS-based time-resolved screening of the conversion from primed to naive pluripotency in hESCs provides a first roadmap of the many changes underlying this transition. The untargeted MS-based approach, as used in our study, not only revealed new hPTM marker candidates in both mouse and human, but also provided combinatorial information, generating a more comprehensive picture of epigenetic changes occurring during the transition of primed hESCs towards the naive state. Ultimately, our findings emphasize that to accurately define the cellular ground state, complementary techniques, such as those capturing the histone code will be critical in the future.

## Methods

### Culture of human embryonic stem cells

Primed hESC line H9 (46, XX) was cultured feeder free on Matrigel (Corning)-coated wells for 4–5 days and refreshed daily with Essential 8 medium (Thermo Fisher Scientific). Passaging was performed using Versene solution (Thermo Fisher Scientific) for 3 minutes, using a cell scraper (VWR). Primed hESC cultures were expanded into 4 separate replicate cultures. Each replicate was subsequently converted to the naive state by applying enhanced naive human stem cell medium (ENHSM, also referred to as WIS-NHSM conditions) [47% DMEM/F12 Medium (Thermo Fisher Scientific), 47% Neurobasal medium (Thermo Fisher Scientific), 0.5% knockout serum replacement (KSR, Thermo Fisher Scientific), 0.06% of a 10% Human Serum Albumin Solution (Thermo Fisher Scientific), 1% non-essential amino acids (Thermo Fisher Scientific), 1% l-glutamine (Thermo Fisher Scientific), 1% penicillin/streptomycin (Thermo Fisher Scientific), 0.005 mM β-mercaptoethanol (Thermo Fisher Scientific), 0.7% sodium pyruvate (Thermo Fisher Scientific), 2% B27 supplement (Thermo Fisher Scientific), 1% N2 supplement (Thermo Fisher Scientific), 0.2% Defined Lipid Concentrate (Thermo Fisher Scientific), 50 µg/ml Ascorbic Acid (Sigma-Aldrich), 1 µM PD0325901 (Cayman Chemicals), 1.5 µM CHIR99021 (Axon Medchem), 20 ng/ml Human Leukaemia Inhibitory Factor (hLIF, Sigma-Aldrich), 2 µM BIRB (Axon Medchem), 5 µM SP600125 (R&D systems Europe), 5 µM Gö (Sigma-Aldrich), 20 ng/ml Activin A (R&D Systems Europe), 10 µM ROCKi (Enzo Life Sciences, only supplemented during feeder free passaging, replaced by ENHSM medium without ROCKi the following day), 12.5 µg/ml insulin (Sigma-Aldrich), 5 µM Inhibitor of Wnt Response 1 (IWR1, Sigma-Aldrich)]^20^. Passaging of hESCs in naive conditions was performed using TrypLE solution (Thermo Fisher Scientific) for 5 minutes, followed by trituration and neutralization with basal ENHSM medium (without small molecules). Cells were collected for histone analysis at passages P0 (Primed hESCs), P3, P6, P9 and P12. At P0 and P12 cells were seeded onto fresh Matrigel-coated plates and grown for 3–4 days for immunofluorescence analysis. RNA was extracted at similar timepoints for qPCR analysis.

### Immunofluorescence

Immunostaining of hESCs was performed as previously described^44^. Briefly, hESCs grown on glass coverslips were fixed with 4% paraformaldehyde (PFA, Sigma-Aldrich) for 20 minutes at room temperature (RT). Cells were then permeabilized with 0.1% Triton X-100 (Sigma-Aldrich) in 1X phosphate buffered saline (PBS) for 8 minutes, washed in PBS and blocked in 10% fetal calf serum (FCS, Sigma-Aldrich) and 0.05% Tween-20 (Sigma-Aldrich) in PBS for 1 hour at RT. Subsequently, the samples were incubated with primary antibodies, mouse anti-OCT3/4 (SC-5279, Santa Cruz, 1:200) and rabbit anti-NANOG (500-P236, Peprotech, 1:200), diluted in 0.05% bovine serum albumin (BSA) in PBS overnight at 4 °C. For the secondary antibodies, Alexa Fluor 594 donkey anti-mouse (AB150105, Abcam, 1:500) and Alexa Fluor 488 donkey anti-rabbit (AB150073, Abcam, 1:500) were used, diluted in 0.05% BSA in PBS and applied for 1 hour at RT. Samples were counterstained with 5 µg/ml DAPI solution (Thermo Fisher Scientific) and mounted on glass microscope slides in ProLong Gold Antifade mounting medium (Thermo Fisher Scientific).

### RNA extraction and qPCR

Primed and naive hESCs from four replicates were collected using Versene and TrypLE solution, respectively. RNA extraction and qPCR were performed as previously described^44^. Briefly, after centrifugation, cell pellets were lysed in TRIzol reagent (Thermo Fisher Scientific), followed by mRNA isolation and purification (RNeasy Mini Kit, Qiagen). Synthesis of single strand cDNA (iScript Advanced cDNA Synthesis Kit, Bio-Rad) was followed by determination of cDNA concentrations with the single strand cDNA Qubit Broad Range Assay kit (Thermo Fisher Scientific). qPCR was performed using the iTAQ Universal SYBR Green Supermix (Bio-Rad) on a Lightcycler 480 instrument (Roche). Amplification steps were programmed to be 1 minute at 95 °C, followed by 95 cycles of 15 seconds at 95 °C and 1 minute at 60 °C. Primer sequences are given in Supplementary Table S6.

Data analysis was performed using the qBasePLUS v3.0 software (Biogazelle). QBase Plus is a semi-automated qPCR analysis software which combines data management, accurate normalization and correct error propagation. Briefly: Cq values that exceeded a 0.5 difference compared to others within triplicates were excluded. Amplification efficiencies were calculated separately, using the LinRegPCR software tool and manually included. Using the gene-specific amplification efficiencies, averaged triplicate values were transformed to linear relative quantities. Next, reference gene normalization was performed according to the most optimal amount of the most stable reference genes over all samples, as determined by the inherent geNorm analysis module: *RPL13A*, *YWHAZ* and *TBP*. Normalized relative quantities of both primed and naive biological replicate samples were plotted as boxplot graphs in GraphPad Prism v6.01. Statistical analysis of gene expression differences between all naive and primed samples was performed using unpaired Student’s t-tests in GraphPad Prism v6.01.

### Histone sample preparation

After assessment of the suitability of different extraction protocols^45^, nuclei were isolated from frozen cell pellets by resuspension in hypotonic lysis buffer (HLB). First, 2.10^6^ cells were resuspended in 400 µl of HLB (10 mM Tris-HCl pH 8.0, 1 mM KCl, 1.5 mM MgCl2) supplemented with 1 mM DTT, Halt protease and phosphatase inhibitor cocktail EDTA-free (788441, Thermo Fisher, 1 mL of cocktail for 100 mL of buffer) and phosphatase inhibitor cocktails II and III (P5726 and P0044, Sigma-Aldrich, 1 mL of cocktail for 100 mL of buffer) and incubated for 30 minutes on a rotator at 4 °C. Subsequently, the nuclei were pelleted and the supernatant was discarded. The pellet was resuspended in 250 µl of 0.4 N HCl and incubated for 30 minutes on a rotator at 4 °C. The histones were precipitated with 33% trichloroacetic acid on ice for 30 minutes. The amount of histones which corresponds to 400,000 cells was quantified by gel-electrophoresis on a 18% TGX gel (Biorad). The remaining purified histones (7.6 µg) of each sample were vacuum dried, and propionylated as previously described^46–48^. Briefly, histones were dissolved in 20 µL 1 M triethylammonium bicarbonate (TEAB) buffer, pH 8.5. Next, 20 μL of propionylation reagent (propionic anhydride: 2-propanol 1:80 (v/v)) was added, for an incubation of 30 minutes at RT. This was followed by adding 20 µl milliQ water (Merck Millipore) for 30 minutes at 37 °C. Histones were then digested overnight at 37 °C using trypsin (at an enzyme/histone ratio of 1:20 (m/m)) in 500 mM TEAB, supplemented with CaCl2 and ACN to a final concentration of 1.0 mM and 5%, respectively. Subsequently, the derivatization reaction was carried out once more, to cap peptide N-termini. Aspecific overpropionylation at serine (S), threonine (T) and tyrosine (Y) was reversed by resuspending the vacuum dried sample in 50 μL 0.5 M NH2OH and 15 μL NH4OH at pH 12 for 20 minutes at RT, after which 30 μl of 100% formic acid (FA) was added.

### Liquid chromatography and mass spectrometry analysis

Liquid chromatography and mass spectrometry analysis was performed as previously described^31^. In short, the 9 μl injection on-column contained 1.5 μg histones and 50 fmol Beta-Galactosidase (Sciex)/MPDS (Waters) internal digest standards in 0.1% FA. Quality control (QC) samples were made by mixing 1 μl of each sample. Peptides were analyzed using low pH reverse phase gradient on the NanoLC™ 425 system operating in microflow mode, coupled to a Triple TOF™ 5600 mass spectrometer (AB SCIEX, Concord, Canada). A Triart C18 150 × 0.3 mm column (YMC) was used at a 5 μL/minute flow rate (0.1% FA with 3% DMSO) with a 60 minute gradient from 3–55% ACN in 0.1% FA, for a total run time of 75 minutes per sample. The sample list was randomized and interspersed with QC injections. Each cycle consisted of one full MS1 scan (m/z 400–1250) of 250 ms, followed by MS2 data-dependent trigger events (m/z 65–2000, high sensitivity mode). A maximum of 10 candidate ions (charge state +2 to +5) exceeding 300 cps were monitored per cycle and excluded for 10 s, with an accumulation time of 200 ms and using a rolling collision energy (CE) with a spread of 15 V. Cycle time was 2.3 s, in order to have 10 to 12 data points per LC peak.

### Mass spectrometry data analysis

For untargeted screening of all relevant hPTMs and in order to overcome ambiguity of annotation, we performed our data analysis according to an established method^32^, which we have previously applied^31^. Briefly, raw data from all runs were imported and aligned in Progenesis QIP 3.0 (Nonlinear Dynamics, Waters) for feature detection. Feature detection was manually validated for all annotated histone features to resolve isobaric near-coelution (*.Archive file available upon request). For identification, per feature three tandem MS (MSMS) spectra closest to the elution apex were selected and exported for searches using Mascot 2.6 (Matrix Science). Three types of searches are always performed: (i) a standard search for the identification of non-propionylated standards (Beta-Galactosidase and MPDS); (ii) an error tolerant search for the identification of all the proteins in the sample; (iii) five sequential searches with six different PTM sets for the identification of modified histone peptides (Supplementary Table S7). The following search criteria were set to identify the standards: trypsin as digestion enzyme, up to one missed cleavage allowed, peptide mass tolerance of 10 ppm and fragment mass tolerance of 50 ppm. For the error tolerant search and the sequential searches, the same criteria were used with exception of ArgC (only cleaved after arginine residues) as digestion enzyme.

The PTM sets used for sequential searching were determined as described previously^31^: to identify proteins in the sample, a default search without biological modifications was executed using a complete (Human) Swissprot database (downloaded from Uniprot on 18/12/2018 and supplemented with contaminants from the cRAP (common Repository of Adventitious Proteins) database (http://www.thegpm.org/crap/) and sequences of spiked standard proteins). From these proteins a FASTA database was generated and all curated PTMs were retrieved from SwissProt. For each feature, all candidate modified peptides in this database were determined based on their MS1 mass. Based on the frequency of each PTM (combination) with respect to the features and modified peptide candidates, the most abundant candidate sets of PTM combinations were selected sequentially. With the inclusion of more sequential searches, the percentage (comprehensiveness) of all considered modified peptide candidates increases. The five sequential searches performed here have a comprehensiveness of 96% of all possible explanations for the MS1 precursor masses found in the combined experiment (Supplementary Table S7). Per search, the top 10 highest scoring (above threshold) annotations per MSMS were reimported into Progenesis QIP. For each feature, all identifications from all searches, from all MSMS spectra were exported with the “Export all Identifications” option of Progenesis QIP. All annotations were analyzed using Python to determine per individual modified peptide isoform if (i) it is biologically modified, (ii) these biological modifications are curated (i.e. are known to exist in curated literature/databases), (iii) this annotation was made on more than one MSMS spectrum (belonging to the same feature) and thus is reproducible. Subsequently, all labeled annotations were classified based on their reproducibility and ambiguity (more than one curated annotation above score threshold) and manually curated by an expert in some cases. Importantly, only previously reported PTMs were retained in this workflow. Protein standardized abundance profiles were created in Progenesis QIP. Here, the quantifiable proteins are grouped according to the similarity between their expression profiles by a correlation analysis. Subsequently, the normalized abundances of all histone precursors were exported from Progenesis QIP for further analysis. PCA plots were generated using ClustVis^49^.

To date the only measurement that allows for direct comparison of single hPTM is RA. As described previously^48^, this is the ratio of the peak area for peptides containing the hPTM of interest, divided by the sum of the peak areas representing the total pool of this peptide. Thus, for each peptide, the RA of each individual hPTM $i$ present on this peptide was calculated as $\frac{{\sum }^{}({\rm{intensities}}\,{\rm{of}}\,{\rm{peptidoforms}}\,{\rm{containing}}\,{\rm{hPTM}}\,i)}{{\sum }^{}({\rm{intensities}}\,{\rm{of}}\,{\rm{all}}\,{\rm{peptidoforms}})}$. Using a standard ANOVA test, a *p*-value was calculated for each hPTM to examine if the passages had a significant effect when considered as a factor. Furthermore, a pairwise *t*-test between each passage of each hPTM was performed to determine which passages introduced a significant difference in the RA of each individual hPTM. For the comparison between mouse and human, the log fold changes of the RA of common hPTMs were retained for creating the scatter plot. K36/K37 were joined together, because resolving both is not trivial in MS.

## Supplementary information

Supplementary Information

Supplementary Table S2

Supplementary Table S3

Supplementary Table S4

Supplementary Table S5

## Data Availability

The mass spectrometry proteomics data have been deposited to the ProteomeXchange Consortium via the PRIDE^50^ partner repository with the dataset identifier PXD013067 and 10.6019/PXD013067.
