# Supplementary material for: Untargeted histone profiling during naive conversion uncovers conserved modification markers between mouse and human
Source: Sci Rep. 2019 Nov 21;9:17240. doi: 10.1038/s41598-019-53681-6 (PMC6872658; doi:10.1038/s41598-019-53681-6)
Supplement: Supplementary file 1 — Supplementary Information [file 41598_2019_53681_MOESM1_ESM.pdf]

Supplementary Information

Untargeted histone profiling during naive conversion uncovers conserved modification markers between mouse and human.

Laura De Clerck[1,4], Jasin Taelman[2,4], Mina Popovic[2], Sander Willems[1], Margot Van der Jeught[2], Björn Heindryckx[2], Petra De Sutter[2], Hendrik Marks[3], Dieter Deforce[1,5], Maarten Dhaenens[1,5,*]

[1] ProGenTomics, Laboratory of Pharmaceutical Biotechnology, Ghent University, Ottergemsesteenweg 460, 9000 Ghent, Belgium

[2] Ghent-Fertility and Stem Cell Team (G-FaST), Department for Reproductive Medicine, Ghent University Hospital, Corneel Heymanslaan 10, 9000, Ghent, Belgium.

[3] Department of Molecular Biology, Faculty of Science, Radboud University, Radboud Institute for Molecular Life Sciences (RIMLS), 6525GA Nijmegen, the Netherlands

[4] These authors contributed equally to this work.

[5] These authors also contributed equally to this work.

[*] Correspondence: maarten.dhaenens@ugent.be

Supplementary figures

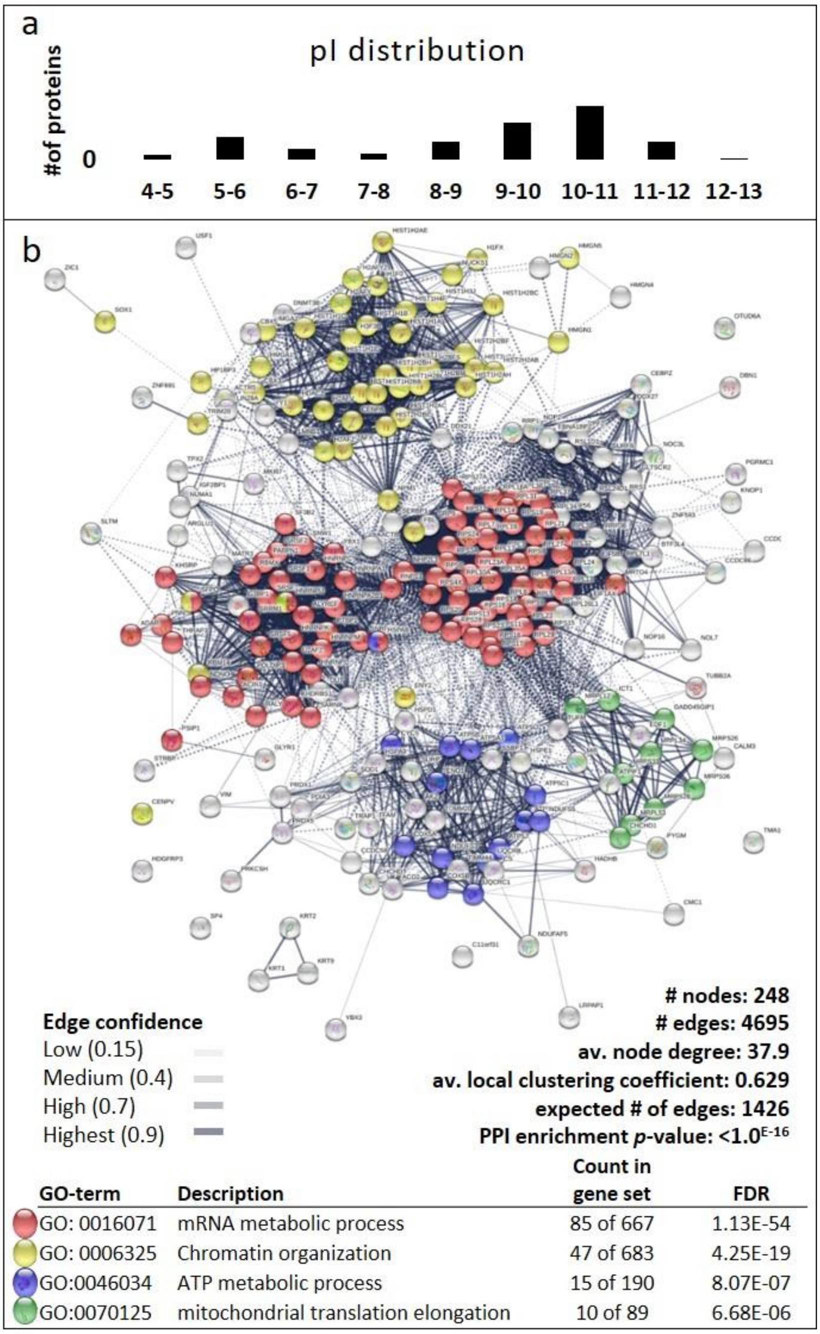

Supplementary Figure S1. Functional analysis of the acid extractome.

250 proteins were (co-)extracted using acid extraction (i.e. acid extractome). **(a)** This subset of the proteome is extremely basic, as seen by their pI distribution calculated using Compute pI/Mw Expasy tool (SIB). **(b)** To understand the identity of this unique subset of the proteome, we subjected these proteins to an association analysis using STRING 11.0 [1,2]. Briefly, the STRING database aims to collect, score and integrate all publicly available sources of protein–protein interaction information, and to complement these with computational predictions. Its goal is to achieve a comprehensive and objective

global network, including direct (physical), as well as indirect (functional) interactions. The 248 proteins were subjected to a confidence mode analysis, leading to a network where the thickness of the line indicates the degree of confidence prediction of the interaction (Edge confidence). All active interaction sources where considered for this network, i.e. text mining, experiments, databases, co-expression, neighborhood, gene fusion and co-occurrence. The average node degree indicates the average number of interactions (at the score threshold) a protein has within the network. The clustering coefficient is a measure of how connected the nodes in the network are. Highly connected networks have high values. The expected number of edges indicates the number of edges anticipated if the nodes were to be selected at random. A small protein-protein interaction (PPI) enrichment *p*-value indicates that the nodes are not random and that the observed number of edges is significant. The confidence of the association network of the acid extractome has a PPI enrichment *p*-value < 1.0E-16, showing the tight functional relationship of the acid extractome. The network was subjected to k-means clustering with 5 clusters. By isolating single proteins from each cluster and examining their function, four GO terms were established that roughly correspond to these clusters: mRNA metabolic process (FDR=1.13E-54), chromatin organization (FDR=4.25E-19), ATP metabolic process (FDR=8.07E-07) and mitochondrial translation initiation (FDR=6.68E-06). These GO terms were used to color the network.

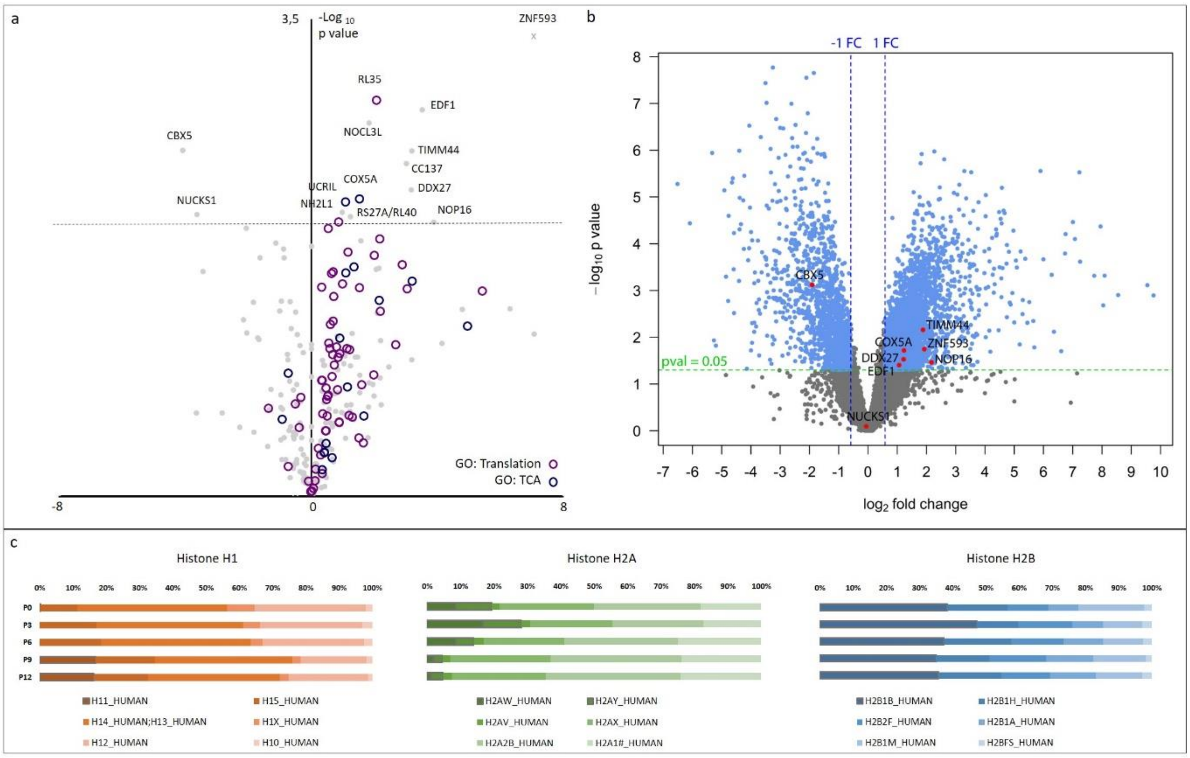

Supplementary Figure S2. Protein and histone variant abundance in the acid extractome.

(a) Volcano plot with the $\log_{10}$ fold (P12/P0) changes depicted in the x-axis. Proteins with a $\log_{10}$ *p*-value < 0.01, i.e. above the dotted line, were labelled with their Uniprot identifier. ZNF593 was artificially set at 8-fold change as it was undetectable in P0 (x). Proteins associated to the GO term

“Translation” are highlighted in purple, proteins associated with the GO term “citric acid cycle (CAC) and respiratory electron transport” are highlighted in dark blue. **(b)** Volcano plots show $\log_2$ fold change of microarray gene expression data from naive (5iLA) versus primed hESCs (according to Theunissen et al., 2014, Cell Stem Cell) and their significance as $-\log_{10}$ *p*-value. Genes that matched proteins with a *p*-value <0.01 in (a) were colored red and named. FC = fold change. Pval = *p*-value. **(c)** Normalized abundance of histone variants of H1, H2A and H2B in the acid extractome throughout the conversion. The differential variants are highlighted in a grey box. Histone H1.1 (ANOVA *q*-value<8.66E-10) is a linker H1 histone that partially replaced other H1 variants at P9 and P12. H2AW (ANOVA *q*-value=4.51e-8) and H2AY (ANOVA *q*-value=1,40e-7) are two forms of macroH2A implemented in X-chromosome inactivation which briefly increased at P3 after which they declined towards P12. Histone H2B1B (ANOVA *q*-value=0,027) briefly increased at P3 and returned to its initial level by P6.

Supplementary tables

Supplementary Table S1. Multiple t-tests on gene expression differences between naive and primed hESCs.

Gene	Significance	P value	Mean1	Mean2	Difference	SE of difference	t ratio	df
**RPL13A**	ns	0.80	1.03	1.00	0.04	0.13	0.27	6
**TBP**	ns	0.43	1.27	0.98	0.29	0.34	0.85	6
**YWHAG**	ns	1.00	1.01	1.01	0.00	0.14	0.00	6
**POU5F1**	ns	0.13	1.82	0.67	1.15	0.67	1.73	6
**NANOG**	ns	0.61	1.15	0.98	0.17	0.31	0.54	6
**DPPA3**	ns	0.05	2.37	0.52	1.85	0.78	2.38	6
**PRDM14**	**	0.01	1.64	0.66	0.98	0.23	4.26	6
**TFCP2L1**	*	0.04	1.32	0.00	1.32	0.50	2.62	6
**ZFP42**	*	0.03	1.71	0.65	1.06	0.37	2.89	6
**OTX2**	*	0.03	0.72	1.49	-0.78	0.29	2.71	6
**ZIC2**	***	0.00	0.09	12.67	-12.58	1.88	6.69	6

Unpaired Student's t-tests were performed comparing NRQ values per gene, from all replicates between naive (passage 12) and primed (passage 0) hESCs. The Benjamini-Hochberg method was used to correct for multiple testing. The false discovery rate was set at 5%. ns = not significant. SE = standard error. df = degrees of freedom. Asterisks represent statistical significance: * *P*<0.05; ** *P*<0.01; *** *P*<0.001.

Supplementary Table S2. Acid Extractome Expression Data of P0, P3, P6, P9 and P12.

See separate Excel File: Table S2.

Supplementary Table S3. Normalized histone peptidoform abundances at P0, P3, P6, P9 and P12.

See separate Excel File: Table S3.

Supplementary Table S4. Pairwise and overall comparison of individual hPTM relative abundances over a time-resolved conversion from naive to primed hESCs.

See separate Excel File: Table S4.

Supplementary Table S5. Differences in histone post translational modifications between naive and primed human embryonic stem cells.

See separate Excel File: Table S5.

Supplementary Table S6. Primer sequences used for qPCR.

**Custom made primer pairs (Thermo Scientific)**		
**Gene**	**Forward**	**Reverse**
**ACTB**	AGAAAATCTGGCACCACACC	TAGCACAGCCTGGATAGCAA
**RPL13A**	CCTGGAGGAGAAGAGGAAAGAGA	TTGAGGACCTCTGTGTATTTGTCAA
**TBP**	CACGAACCACGGCACTGATT	TTTTCTTGCTGCCAGTCTGGAC
**YWHAG**	AGAACCACGATGACCACAGAC	AGGCACAAAAGCGGCAAAG

**Readymade Taqman assays (Thermo Scientific)**	
**Gene**	**Taqman assay ID**
**OTX2**	Hs00222238_m1
**POU5F1**	Hs01895061_u1
**PRDM14**	Hs01119056_m1
**NANOG**	Hs02387400_g1
**TFCP2L1**	Hs01011666_m1
**ZFP42**	Hs00399279_m1
**ZIC2**	Hs00600845_m1

Supplementary Table S7. PTM sets used for Sequential searches.

							**% of spectra (17089)**	**% of annotatable spectra (12306)**
Unannotatable							0.28	/
No variable PTMs							0.43	0.6
Search 1	K-42.01	K-28.03	K-100.02	K-70.04	M-15.99	R-14.02	0.61	0.85
Search 2	K-42.01	K-68.03	K-70.04	K-86.04	R-28.03	Nterm-42.01	0.66	0.91
Search 3	K-42.01	K-42.05	K-68.03	K-100.02	K-86.04	R-0.98	0.67	0.93
Search 4	K-28.03	K-42.05	K-86.04	M-15.99	R-14.02	R-28.03	0.68	0.95
Search 5	K-42.01	K-28.03	K-42.05	K-68.03	K-70.04	R-0.98	0.69	**0.96**

0.98, citrullination; 14.02, methylation; 15.99, oxidation; 27.99, formylation; 28.03, dimethylation; 42.01, acetylation; 42.05, trimethylation; 68.03, crotonylation; 70.04, butyryl / methylation (+ propionyl); 86.04, 2-hydroxyisoMeation; 100.02, succinylation.

References

1. Szklarczyk, D. *et al.* The STRING database in 2017: quality-controlled protein-protein association networks, made broadly accessible. *Nucleic Acids Res.* **45**, D362–D368 (2017).

2. Szklarczyk, D. *et al.* STRING v10: protein–protein interaction networks, integrated over the tree of life. *Nucleic Acids Res.* **43**, D447–D452 (2015).
